# The conclusiveness of trial sequential analysis varies with estimation of between-study variance: a case study

**DOI:** 10.1186/s12874-025-02545-x

**Published:** 2025-04-17

**Authors:** Enoch Kang, James S. Hodges, Yu-Chieh Chuang, Jin-Hua Chen, Chiehfeng Chen, Cochrane Taiwan, Ka-Wai Tam, Kee-Hsin Chen, Wen Hsuan Hou, Tsai-Wei Huang, El-Wui Loh

**Affiliations:** 1 Cochrane Taiwan, Taipei Medical University, Taipei, Taiwan; 2 Evidence-Based Medicine Center, Wan Fang Hospital, Taipei Medical University, No. 111, Section 3, Xinglong Road, Taipei, Taiwan; 3 Institute of Health Policy & Management, College of Public Health, National Taiwan University, Taipei, Taiwan; 4 Division of Biostatistics and Health Data Sciences, School of Public Health, University of Minnesota, Minneapolis, MN USA; 5 Graduate Institute of Data Science, College of Management, Taipei Medical University, Taipei, Taiwan; 6 College of Public Health, National Taiwan University, Taipei, Taiwan; 7 Department of Psychiatry, Taipei City Psychiatric Center, Taipei City Hospital, Songde Branch, Taipei, Taiwan; 8 School of Medicine, College of Medicine, Taipei Medical University, Taipei, Taiwan; 9 Graduate Institute of Data Science, College of Management, Taipei Medical University, Taipei, 110 Taiwan; 10 Research Center of Biostatistics Center, College of Management, Taipei Medical University, Taipei, 110 Taiwan; 11 Biostatistics Center, Wan Fang Hospital, Taipei Medical University, Taipei, 116 Taiwan; 12 Department of Public Health, School of Medicine, College of Medicine, Taipei Medical University, Taipei, Taiwan; 13 Division of Plastic Surgery, Department of Surgery, Wan Fang Hospital, Taipei Medical University, Taipei, Taiwan

**Keywords:** Meta-analysis, Sequential method, Tau-square, Heterogeneity, Required information size, Optimal information size

## Abstract

**Background:**

Trial sequential methods have been introduced to address issues related to increased likelihood of incorrectly rejecting the null hypothesis in meta-analyses due to repeated significance testing. Between-study variance (τ^2^) and its estimate ($\widehat{\tau }$ ^2^) play a crucial role in both meta-analysis and trial sequential analysis with the random-effects model. Therefore, we investigated how different $\widehat{\tau }$ ^2^ impact the results of and quantities used in trial sequential analysis.

**Methods:**

This case study was grounded in a Cochrane review that provides data for smaller (< 10 randomized clinical trials, RCTs) and larger (> 20 RCTs) meta-analyses. The review compared various outcomes between video-laryngoscopy and direct laryngoscopy for tracheal intubation, and we used outcomes including hypoxemia and failed intubation, stratified by difficulty, expertise, and obesity. We calculated odds ratios using inverse variance method with six estimators for *τ*^2^, including DerSimonian-Laird, restricted maximum-likelihood, Paule-Mandel, maximum-likelihood, Sidik-Jonkman, and Hunter-Schmidt. Then we depicted the relationships between $\widehat{\tau }$ ^2^ and quantities in trial sequential analysis including diversity, adjustment factor, required information size (RIS), and *α*-spending boundaries.

**Results:**

We found that diversity increases logarithmically with $\widehat{\tau }$ ^2^, and that the adjustment factor, RIS, and *α*-spending boundaries increase linearly with $\widehat{\tau }$ ^2^. Also, the conclusions of trial sequential analysis can differ depending on the estimator used for between-study variance.

**Conclusion:**

This study highlights the importance of $\widehat{\tau }$ ^2^ in trial sequential analysis and underscores the need to align the meta-analysis and the trial sequential analysis by choosing estimators to avoid introducing biases and discrepancies in effect size estimates and uncertainty assessments.

**Graphical Abstract:**

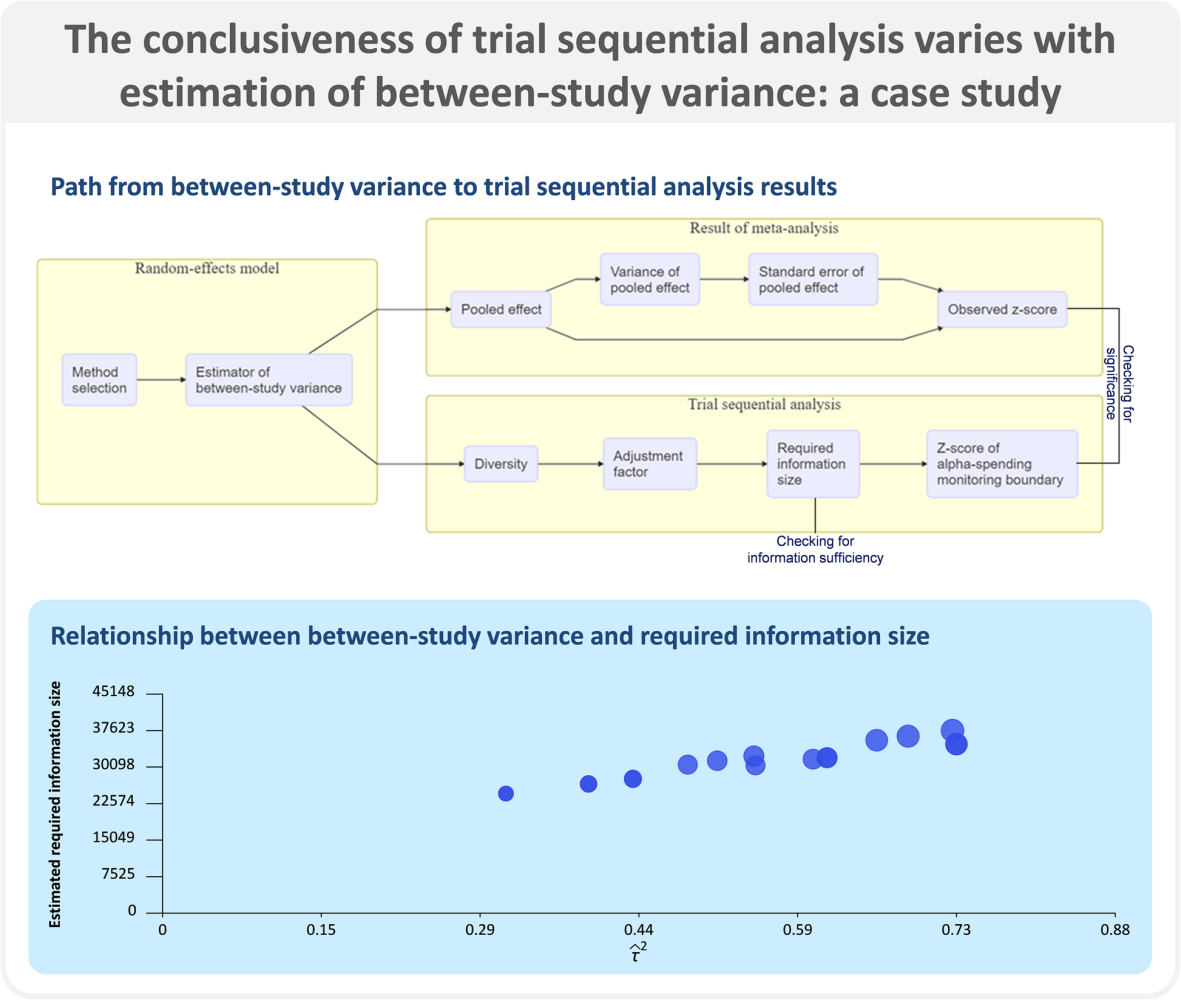

**Supplementary Information:**

The online version contains supplementary material available at 10.1186/s12874-025-02545-x.

## What is new?

### What is already known on this topic

Diversity, a quantity in trial sequential analysis, is related to between-study variance.

### What this study adds

Between-study variance estimates are consistently associated with all quantities in trial sequential analysis, regardless of whether the meta-analysis is small or large.

### How this study might affect research, practice or policy

Consistency between meta-analysis and trial sequential analysis in variance estimators is crucial for integrity and validity.

## Introduction

Cumulative meta-analysis has been introduced as a useful method of identifying intervention benefits early and determining the statistical significance of evidence [1, 2], although challenges such as multiplicity and biased reporting complicate this approach. Repeated significance testing increases the likelihood of rejecting the null hypothesis [3], a concern that can be mitigated by using trial sequential methods. Group sequential analysis and cumulative *z*-curve modeling offer methods for monitoring trial progress and determining when to terminate a trial based on accumulating evidence [4–6]. These methods have been extended to meta-analysis for controlling falsely significant results (Type I errors, *α*) due to biases or random errors from repeated testing [1–3, 7–9]. Because of the sequential method's capacity for error correction in aggregating evidence, several articles in diverse medical specialties have introduced, endorsed, and apply trial sequential analysis [10–15]. These references frequently underscore the importance of identifying key quantities in computing for the method, which include the required information size (RIS), diversity (*D*^2^), and *α*-spending boundaries. Evidence is considered confirmed when the cumulative z-score crosses the α-spending monitoring boundaries [3, 10, 16]. The *α*-spending boundaries provide a threshold of significance by allocating the overall *α* among the cumulative analyses. This adjusted threshold guides researchers in determining whether the cumulative evidence reaches statistical significance. Hence, it is imperative to understand better not only the above quantities but also the various factors that are used in computing these quantities and thus may affect the results of a trial sequential analysis.

Between-study variance (*τ*^2^) is an important quantity for both the meta-analysis and the trial sequential analysis in a random-effects model. It is well-known that the estimate *τ*^2^, $\widehat{\tau }$ ^2^, is combined with within-study variance (denoted ν_i_) to produce a weight for each study ${W}_{random.i}=\frac{1}{ ({\nu }_{i}+{\widehat{\tau }}^{2})}$, through which $\widehat{\tau }$ ^2^ influences the estimated pooled effect (denoted $\widehat{\theta }$_random_) and it variance (denoted $\widehat{\nu }$_random_) [17]. The standard error and z-score for the pooled result are further derived based on $\widehat{\tau }$ ^2^. Between-study variance is also a critical quantity in trial sequential analysis, as it affects the RIS. The RIS for a meta-analysis using the random-effects model ($\widehat{RIS}$_random_) can be expressed as follows:

1 $$\widehat{RIS}_{random}=4*\frac{{{(Z}_{\alpha /2}+{Z}_{\beta })}^{2}*{\widehat{\nu }}_{random}}{{\mu }_{random}^{2}}$$

where

*α* is the predefined overall probability of a false positive.

*β* is the predefined overall probability of a false negative.

*μ*^*2*^ is the expected effect.

$\widehat{\nu }$_*random*_ is the variance of pooled effects of the meta-analysis in random-effects model.

*Note:*

1. $\widehat{\nu }$_*random*_ can be obtained from $\frac{1}{\sum_{i=1}^{j}{W}_{random.i}}$.

2. $\widehat{RIS}_{random}$ here is also unadjusted required information size ($\widehat{RIS}$_*unadjusted.random*_).

Considering heterogeneity (also known as between-study variance) in meta-analysis, the inclusion of an adjustment factor (AF) becomes imperative for estimating the RIS ($\widehat{RIS}$). Diversity (*D*^2^) has been proposed as a measure and as a foundational quantity for the AF and the adjusted RIS, which is the unadjusted RIS multiplied by the AF [18]. *D*^2^ is the total relative variance when changing from a pooled analysis using the common-effect model to a meta-analysis using the random-effects model. The $\widehat{D}$ ^2^ can be expressed as follows:

2 $$\widehat{D}^{2} =\frac{{\widehat{\nu }}_{random}- {\nu }_{fixed}}{{\widehat{\nu }}_{random}}$$

where

*ν*_*fixed*_ is the variance of pooled effects of the meta-analysis in fixed-effect model (also known as common-effect model).

$\widehat{\nu }$_*random*_ is the variance of pooled effects of the meta-analysis in random-effects model.

An alternative expression of $\widehat{D}$ ^2^ can highlight the role of the estimated between-study variance in the calculation, as follows:

3 $$\widehat{D}^{2}= \frac{1}{{\widehat{\tau }}^{2}}*({\widehat{\tau }}^{2}+ \frac{{\widehat{\tau }}^{2}* {\nu }_{fixed}}{ {\widehat{\nu }}_{random} - {\nu }_{fixed}})$$

where

*ν*_*fixed*_ is the variance of pooled effects of the meta-analysis in fixed-effect model (also known as common-effect model).

$\widehat{\nu }$_*random*_ is the variance of pooled effects of the meta-analysis in random-effects model.

${\widehat{\tau }}^{2}$ is estimated between-study variance of the meta-analysis in random-effects model.

Then, the adjustment factor of the $\widehat{RIS}$ (denoted as $\widehat{AF}$) can be derived using *D*^2^, as expressed $\widehat{AF}$ = $\frac{1}{{(1-\widehat{D}}^{2})}$. Since $\widehat{\tau }$ ^2^ is a crucial quantity in the random-effects model, it impacts the estimates of diversity ($\widehat{D}$ ^2^), adjustment factor ($\widehat{AF}$), and the adjusted RIS ($\widehat{RIS}$_adjusted_). The estimate of *τ*^2^, $\widehat{\tau }$ ^2^, is crucial for both meta-analysis and trial sequential analysis using a random-effects model. Previous studies have pointed out the importance of between-study variance within trial sequential analysis using a random-effects model, but the influence of different estimators of between-study variance on various quantities in such an analysis warrants further discussion [3, 18, 19].

The RIS plays a crucial role in trial sequential analysis, serving as both a threshold for the methodological and clinical establishment of evidence, and as the foundation for calculating *α*-spending boundaries [3, 7, 10]. The concept of RIS originated from the optimal information size, drawing upon quantities such as expected effect size, variance, and predetermined *α* and Type II errors (*β*) [3, 7]. The RIS can be obtained using the expected effect size, the variance of the pooled model, and predetermined *α* and *β*.

In sequential meta-analysis using the random-effects model, $\widehat{\tau }$ ^2^ plays a critical role due to its impact on both the observed cumulative z-score and $\widehat{D}$ ^2^. An estimator for the between-study variance can be derived by various approaches, e.g., the method of moments, maximum likelihood, and the model error variance estimator [20]. Owing to differences between these different estimators of between-study variance [21–23], both $\widehat{D}$ ^2^ and $\widehat{RIS}$ can also differ depending on the estimator chosen. This case study aimed to enhance understanding of how the choice of $\widehat{\tau }$ ^2^ influences quantities used in trial sequential analysis in random-effects model.

## Methods

This case study aimed to elucidate the influence of $\widehat{\tau }$ ^2^ in trial sequential analysis by illustrating its relationship with the observed cumulative z-score, estimated diversity ($\widehat{D}$ ^2^), adjustment factor ($\widehat{AF}$), required information size ($\widehat{RIS}$), and the z-score of the *α*-spending boundaries. The registration for this study can be accessed through the Open Science Framework (https://osf.io/czstm). In addition to the registration, we add an example to demonstrate the relationship between $\widehat{\tau }$ ^2^ and the conclusiveness of evidence. This study was proposed to depict the aforementioned relationship across various study sizes, considering the presence or absence of significant heterogeneity. Consequently, we intended to identify a Cochrane review that includes both smaller and larger meta-analyses, including cases of both significant and non-significant heterogeneity based on the specified criteria as follows: (1) systematic reviews with meta-analyses having < 10 randomized clinical trials (RCTs) and > 20 RCTs, (2) outcomes with varying levels of between-study variance, and (3) data availability.

### The case study used here

A systematic review conducted by Hansel et al. (2022) fulfilling the criteria outlined above was chosen for use in this study [24]. Specifically, this study used data from the Cochrane review that investigated the comparative effects of video-laryngoscopy and direct laryngoscopy in adults undergoing tracheal intubation. The Cochrane review includes outcomes with or without significant heterogeneity according to the *P*-value from the *χ*^2^-test for heterogeneity (*P* < 0.1) based on the DerSimonian-Laird method (DL). The low power of the *χ*^2^-test for heterogeneity in meta-analyses with small or few studies means that a non-significant result cannot be taken as evidence of no heterogeneity, which justifies sometimes using a *P*-value of 0.10 to assess statistical significance instead of the conventional 0.05 [25]. We extracted four outcomes from the review, including a small meta-analysis comparing hypoxemia using seven RCTs without significant heterogeneity (*P*-value = 0.68), failed intubation in difficult cases using nine RCTs with significant heterogeneity (*P*-value = 0.0993). We also analyzed larger meta-analyses focusing on failed intubation using 48 and 62 RCTs, categorized by practitioner expertise without significant heterogeneity (*P*-value = 0.65) and by obesity status with significant heterogeneity (*P*-value = 0.0003), respectively (Supplementary file 1). We also selected data on failed intubations comparing hyper-angulated video laryngoscopy and direct laryngoscopy in patients with difficult cases to provide a clear example of evidence conclusiveness using $\widehat{\tau }$ ^2^.

### Methods for estimating between-study variance

For each of the above outcomes, we estimated the odds ratio using inverse variance. The present study does not include the results from Mantel–Haenszel and Peto’s pooling methods due to discouragement about their applicability, despite the intention to incorporate them in our initial registration and early analyses. To understand the role of $\widehat{\tau }$ ^2^ in trial sequential analysis, we considered six estimators for *τ*^2^: DL [26], restricted maximum-likelihood (REML) [27], Paule-Mandel [28], maximum-likelihood [27], Sidik-Jonkman [29], and Hunter-Schmidt [30]. Equations for the estimators are given in Supplementary file 2.

### Methods for trial sequential analysis

Supplementary file 3 presents detailed formulae for the trial sequential analysis methods used in this study [3, 7, 18]. The main formulae are for $\widehat{D}$ ^2^, $\widehat{AF}$, $\widehat{RIS}$, and the z-score of the *α*-spending boundaries. Briefly, $\widehat{\tau }$ ^2^ is calculated as part of using the random-effects model and influences the variance of the pooled estimate, which directly impacts both $\widehat{D}$ ^2^ and $\widehat{RIS}$. Subsequently, through $\widehat{D}$ ^2^ and $\widehat{RIS}$, $\widehat{\tau }$ ^2^ and the variance of the pooled estimate also affect $\widehat{AF}$ and the z-score of the *α*-spending boundaries. We describe variability of those quantities using range and quartile coefficient of variation (QCV) [31, 32].

### Software

All analyses were done using *R* version 4.2.2, specifically the function `metabin()` of the package *meta* (version 7.0–0) to perform meta-analysis and the function `DoTSA()` of the package *smiles* (version 0.1–0) to perform trial sequential analysis [33–36]. The R package *RTSA* has been endorsed by Copenhagen Trial Unit for performing trial sequential analysis [37]; however, it has limited options for estimating the between-study variance. Therefore, we opted the R package *smiles*, which supports all the estimators of between-study variance mentioned in the R package *meta*. When given the same inputs, the TSA software (version 0.9.5.10 Beta, Copenhagen Trial Unit, Copenhagen University Hospital – Rigshospitalet, Denmark) and the function `DoTSA()` of the R package *smiles* returned the same values for the RIS. Supplementary file 4 shows one example using hypoxaemia data with the DL estimator, *α* = 0.05, *β* = 0.2, relative risk reduction 20%. Supplementary file 5 gives the R code used for this study. Supplementary file 6 summarizes the quantities computed in the trial sequential analyses.

## Results

Supplementary file 7 shows the sequence of calculations through which the estimated between-study variance ($\widehat{\tau }$ ^2^) impacts the results of both a meta-analysis and a trial sequential analysis. As the observed cumulative *z*-score of a meta-analysis is commonly compared to the *z*-score of an *α*-spending monitoring boundaries in a trial sequential analysis, in the present study we focus on exploring the relationship between $\widehat{\tau }$ ^2^ and the observed cumulative *z*-score of the meta-analysis, and on the relationship between $\widehat{\tau }$ ^2^ and the calculations done in parallel with $\widehat{D}$ ^2^, $\widehat{AF}$, $\widehat{RIS}$, and the z-score of the *α*-spending boundaries.

### Relationship between $\widehat{{\varvec{\tau}}}$^2^ and $\widehat{{\varvec{D}}}$^2^

**Fig. 1:**
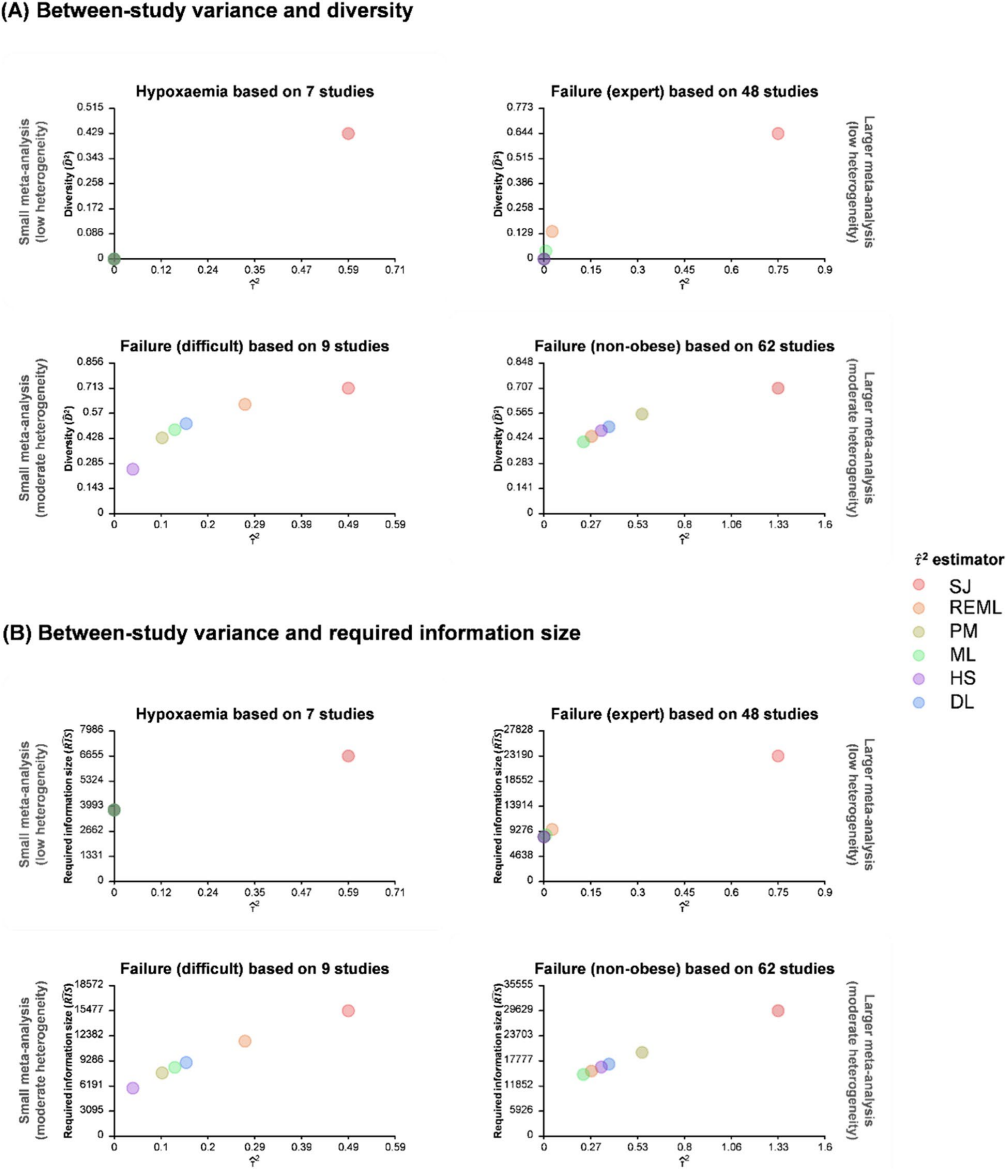
Scatter plots of (**A**) diversity ($\widehat{D}$ ^2^) versus estimated between-study variance ($\widehat{\tau }$ ^2^) and (**B**) required information size ($\widehat{RIS}$) versus estimated between-study variance ($\widehat{\tau }$ ^2^), for smaller meta-analysis without significant heterogeneity, smaller meta-analysis with significant heterogeneity, larger meta-analysis without significant heterogeneity, and larger meta-analysis with significant heterogeneity. DL, DerSimonian-Laird estimator; HS, Hunter-Schmidt estimator; ML, Maximum-likelihood estimator; PM, Paule-Mandel estimator; REML, Restricted maximum-likelihood estimator; SJ, Sidik-Jonkman estimator

Figure 1A shows that $\widehat{D}$ ^2^ increases as $\widehat{\tau }$ ^2^ increases; the relationship appears to follow a logarithmic curve because $\widehat{D}$ ^2^ is between 0 and 1. This increasing trend is consistent across the four outcomes, while $\widehat{D}$ ^2^ seems to be sensitive to the selection of $\widehat{\tau }$ ^2^ due to its range between 0 to 0.43, 0.25 to 0.71, 0 to 0.68, and 0.40 to 0.71 in the four outcomes. In meta-analysis with non-significant heterogeneity, the QCV of $\widehat{D}$ ^2^ achieved 100% among the six between-study variance estimators (Supplementary file 8). Because the Sidik-Jonkman approach produces the largest $\widehat{\tau }$ ^2^ across the four outcomes, the red points for Sidik-Jonkman usually have the largest $\widehat{D}$ ^2^.

### Relationship between $\widehat{{\varvec{\tau}}}$^2^, the adjustment factor and the required information size

The diversity-based adjustment factor ($\widehat{AF}$_*D*_^2^) increases linearly as $\widehat{\tau }$ ^2^ increases (Supplementary file 8), and the relationship between $\widehat{\tau }$ ^2^ and the diversity-adjusted required information size ($\widehat{RIS}$_*D*_^2^) was also nearly linear (Fig. 1B). These two figures have similar patterns because $\widehat{RIS}$_*D*_^2^ is computed as $\widehat{RIS}$_*unadjusted*_ $\widehat{AF}$_*D*_^2^. However, both quantities are sensitive to the selection of $\widehat{\tau }$ ^2^, even in meta-analyses without significant heterogeneity. $\widehat{AF}$_*D*_^2^ varies from 1 to 1.75, 1.34 to 3.48, 1 to 2.81, and 1.68 to 3.41 across the four outcomes. Similarly, $\widehat{RIS}$_*D*_^2^ varies between 3789 and 6655, 5940 and 15,477, 8254 and 23,190, and 14,588 and 29,629 for the respective outcomes. The QCV ranges between 0 and 16% across the four outcomes.

### Relationship between $\widehat{{\varvec{\tau}}}$^2^ and *α*-spending boundaries

**Fig. 2:**
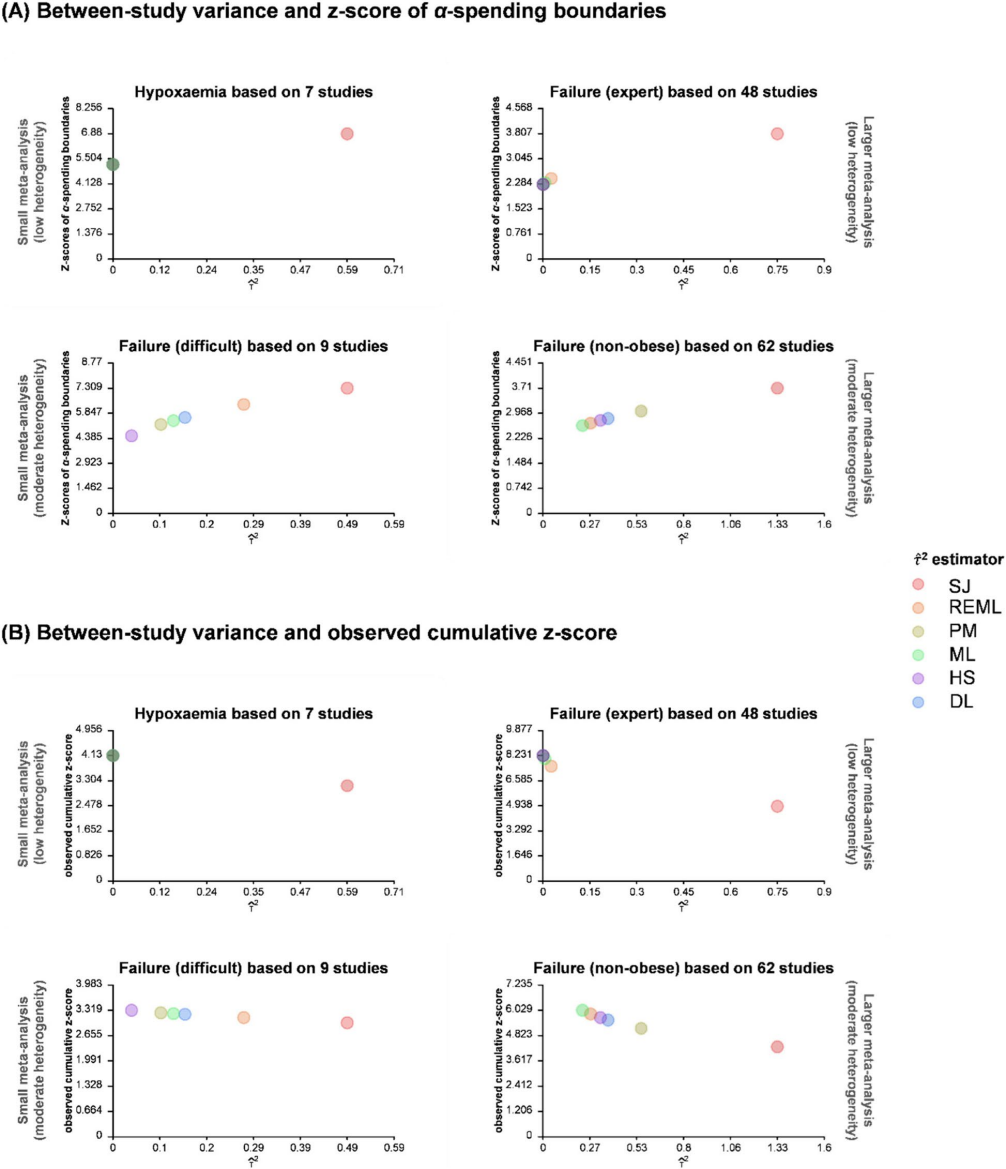
Scatter plots of (**A**) *α*-spending monitoring boundary versus estimated between-study variance ($\widehat{\tau }$ ^2^) and (**B**) observed cumulative z-score versus estimated between-study variance ($\widehat{\tau }$ ^2^), for smaller meta-analysis without significant heterogeneity, smaller meta-analysis with significant heterogeneity, larger meta-analysis without significant heterogeneity, and larger meta-analysis with significant heterogeneity. DL, DerSimonian-Laird estimator; HS, Hunter-Schmidt estimator; ML, Maximum-likelihood estimator; PM, Paule-Mandel estimator; REML, Restricted maximum-likelihood estimator; SJ, Sidik-Jonkman estimator

Figure 2A shows a trend in which, in most panels of the Figure, the *α*-spending boundaries increase linearly as $\widehat{\tau }$ ^2^ increases. The minimum *α*-spending boundary ranges from − 6.88 to − 5.20 (QCV = 0%), from − 7.13 to − 4.53 (QCV = 8%), from − 3.81 to − 2.27 (QCV = 3%), and from − 3.71 to − 2.60 (QCV = 5%) for the four outcomes respectively.

### Relationship between $\widehat{{\varvec{\tau}}}$^2^ and observed cumulative z-score

In Fig. 2B, the primary phenomenon in all four outcomes is a decreasing trend in cumulative z-score as $\widehat{\tau }$ ^2^ increases. The minimum cumulative z-score varies, with a range of − 4.13 to − 3.14 for QCV at 0%, − 3.32 to − 2.99 for QCV at 2%, − 8.23 to − 4.90 for QCV at 4%, and − 6.03 to − 4.29 for QCV at 5%, reflecting the four distinct outcomes. Notably, the Sidik-Jonkman approach seems to yield the largest $\widehat{\tau }$ ^2^ among the estimators irrespective of outcome.

### Evidence conclusiveness by $\widehat{{\varvec{\tau}}}$^2^

**Fig. 3:**
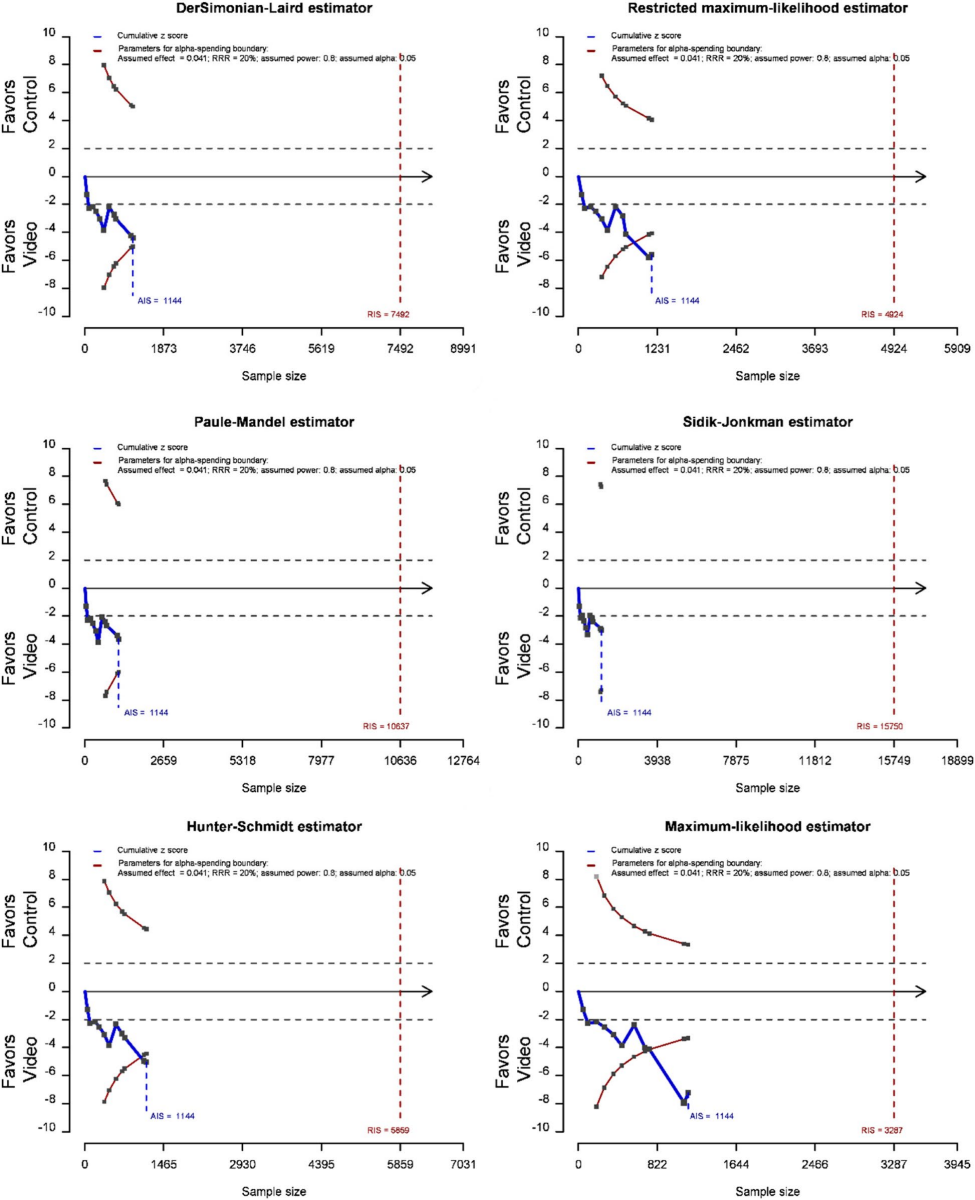
Trial sequential analysis plots using different between-study variance estimators ($\widehat{\tau }$ ^2^) for failed intubations comparing hyper-angulated video laryngoscopy and direct laryngoscopy. DL, DerSimonian-Laird estimator; HS, Hunter-Schmidt estimator; ML, Maximum-likelihood estimator; PM, Paule-Mandel estimator; REML, Restricted maximum-likelihood estimator; SJ, Sidik-Jonkman estimator

Based on data comparing failed intubations between hyper-angulated video laryngoscopy and direct laryngoscopy in patients with difficult cases, the conclusiveness of the evidence depends on the choice of the estimator $\widehat{\tau }$ ^2^. The evidence is conclusive and statistically significant when applying REML, Hunter-Schmidt, and maximum-likelihood estimators (Fig. 3).

## Discussion

This study found distinct patterns showing how the estimator of between-study variance, $\widehat{\tau }$ ^2^, affects both meta-analysis and trial sequential analysis. The profound implications of choosing an estimator for *τ*^2^ are well known [20–23, 38]. These investigations found wide disparities in estimates of *τ*^2^ across diverse scenarios characterized by differing data types (dichotomous and continuous), effect sizes, and levels of true *τ*^2^. For instance, the Sidik-Jonkman approach consistently tends to overestimate *τ*^2^, irrespective of whether the meta-analysis is small or large, and regardless of the type of outcome (dichotomous or continuous) [20, 23]. This phenomenon can also be observed in our study (red points in Figs. 1 and 2).

Importantly, $\widehat{\tau }$ ^2^ is the bedrock of trial sequential analysis using the random-effects model [15]. It is a critical quantity for estimating the RIS, which is essential for establishing a threshold of information sufficiency and for defining *α*-spending boundaries [3, 7, 18]. Typically, adjusting the RIS is necessary when there is between-study variance (*τ*^2^) in a meta-analysis using the random-effects model. The adjustment is fundamentally based on $\widehat{\tau }$ ^2^ and is especially relevant when incorporating diversity ($\widehat{D}$ ^2^) into the adjustment process [18]. The patterns in the figures offer an overview on the crucial role of selecting an estimator of between-study variance in trial sequential analysis due to the impact of $\widehat{\tau }$ ^2^ on $\widehat{D}$ ^2^, $\widehat{AF}$, $\widehat{RIS}$, and the z-score of the *α*-spending boundaries.

### Implications

Given the need to choose an estimator of between-study variance in trial sequential analysis, two practical recommendations emerge. First, consistency between meta-analysis and trial sequential analysis in selecting between-study variance estimators is paramount for maintaining the integrity and validity of the research findings. Inconsistency in these choices can introduce biases and undermine the reliability of conclusions drawn from sequential analyses. For instance, using different between-study variance estimators in the two analytical phases may lead to discrepancies in effect size estimates and uncertainty assessments, impacting the interpretation of results. Therefore, researchers must carefully align these choices of methods across meta-analysis and trial sequential analysis. Secondly, protocol design for synthesizing evidence using sequential methods requires careful consideration of the estimator that is chosen. Therefore, including these choices of methods in the protocol ensures the consistency and rigor necessary for producing trustworthy evidence through sequential synthesis methods.

### Limitations

While this study delineates step-by-step the role of between-study variance at various stages of trial sequential analysis, it is important to acknowledge several limitations. Primarily, the study's scope was constrained by the availability of published data from a Cochrane review, limiting the depth of analysis for certain phenomena. For instance, while this study provides valuable insights into estimated between-study variance ($\widehat{\tau }$ ^2^), further investigation is warranted across diverse scenarios such as different effect sizes, sparse data, and unequal sample sizes. A comprehensive examination of the varying impacts of $\widehat{\tau }$ ^2^ will require exploration through simulation studies. Second, this study's findings rely on between-study variance estimators that are commonly used in meta-analyses and cannot show which estimators are better or worse for trial sequential analysis across different scenarios, underscoring the need for further research in this area. Third, this work performs the role of $\widehat{\tau }$ ^2^ in trial sequential analysis based on dichotomous data, while we know that $\widehat{\tau }$ ^2^ varies by differing data types. Further studies are encouraged to use continuous data to illustrate the relationships and quantities in trial sequential meta-analysis within a random-effects model. Lastly, the study does not explore the relationship between $\widehat{\tau }$ ^2^ and the *β*-spending function, which is a reasonable decision considering its primary focus. However, we contend that $\widehat{\tau }$ ^2^ may have an impact on the *β*-spending function because of its influence on the RIS. Further investigation into how $\widehat{\tau }$ ^2^ affects the *β*-spending function would be valuable.

## Conclusions

This study sheds light on the influence of the estimator of between-study variance in trial sequential analysis, emphasizing the crucial need for predetermined and consistent use of estimation methods for this variance. Besides, trial sequential analysis seems to be sensitive to the $\widehat{\tau }$ ^2^ used to establish its boundaries. This presents a challenge, as the estimation of between-study variance frequently lacks precision, especially in cases with a limited number of studies. By enriching our understanding of these complexities, these efforts will enhance the integrity and utility of trial sequential analysis in informing evidence-based practice and decision-making in healthcare.

## Availability and requirements

Project name: Sequential Method In Leading Evidence Synthesis (SMILES).

Project home page: https://osf.io/td689/.

Operating system: Microsoft Windows.

Programming language: R

Other requirements: R version 4.2.2

License: General Public License.

## Supplementary Information

Supplementary Material 1. Data source, effect estimates, and assessment of heterogeneity, Equations for between-study variance estimators, Equations for quantities used in trial sequential analysis, TSA software and R package *smiles *return the same required information size, R code to perform the sequential analysis in this study, Summary of quantities computed in the trial sequential analyses for this study, Flowchart of the steps in using the random-effect model in trial sequential analysis, Scatter plots of the adjustment factor (AF) versus the estimated between-study variance ($\widehat{{\varvec{\tau}}}$^2^), Descriptive statistics and variability of quantities in the trial sequential analyses.

## Data Availability

We used data from a systematic review conducted by Hansel et al. (2022) [22]. Data in this study are available to other researchers upon reasonable request to corresponding authors C.F.C.

## Abbreviations

AF: Adjustment factor
DL: DerSimonian-Laird
HS: Hunter-Schmidt
ML: Maximum-likelihood
PM: Paule-Mandel
RCT: Randomized clinical trial
REML: Restricted maximum-likelihood
RIS: Required information size
SJ: Sidik-Jonkman

## References

[1] Lau J, Antman EM, Jimenez-Silva J, Kupelnick B, Mosteller F, Chalmers TC. Cumulative meta-analysis of therapeutic trials for myocardial infarction. N Engl J Med. 1992;327(4):248–54. 10.1056/nejm199207233270406. 1614465 10.1056/NEJM199207233270406

[2] Lau J, Schmid CH, Chalmers TC. Cumulative meta-analysis of clinical trials builds evidence for exemplary medical care. J Clin Epidemiol. 1995;48(1):45–57; discussion 9–60. 10.1016/0895-4356(94)00106-z. 10.1016/0895-4356(94)00106-z 7853047

[3] Wetterslev J, Thorlund K, Brok J, Gluud C. Trial sequential analysis may establish when firm evidence is reached in cumulative meta-analysis. J Clin Epidemiol. 2008;61(1):64–75. 10.1016/j.jclinepi.2007.03.013. 18083463 10.1016/j.jclinepi.2007.03.013

[4] Armitage P. Sequential analysis in therapeutic trials. Annu Rev Med. 1969;20:425–30. 10.1146/annurev.me.20.020169.002233. 4893407 10.1146/annurev.me.20.020169.002233

[5] Pocock SJ. Group sequential methods in the design and analysis of clinical trials. Biometrika. 1977;64(2):191–9.

[6] Gordon Lan K, DeMets DL. Discrete sequential boundaries for clinical trials. Biometrika. 1983;70(3):659–63.

[7] Pogue JM, Yusuf S. Cumulating evidence from randomized trials: utilizing sequential monitoring boundaries for cumulative meta-analysis. Control Clin Trials. 1997;18(6):580–93; discussion 661–6. 10.1016/s0197-2456(97)00051-2. 10.1016/s0197-2456(97)00051-2 9408720

[8] Imberger G, Gluud C, Boylan J, Wetterslev J. Systematic Reviews of Anesthesiologic Interventions Reported as Statistically Significant: Problems with Power, Precision, and Type 1 Error Protection. Anesth Analg. 2015;121(6):1611–22. 10.1213/ane.0000000000000892. 26579662 10.1213/ANE.0000000000000892

[9] Imberger G, Thorlund K, Gluud C, Wetterslev J. False-positive findings in Cochrane meta-analyses with and without application of trial sequential analysis: an empirical review. BMJ Open. 2016;6(8): e011890. 10.1136/bmjopen-2016-011890. 27519923 10.1136/bmjopen-2016-011890 PMC4985805

[10] Greco A, Capodanno D. Trial sequential analysis methodology for interpreting meta-analytical findings. Eur J Intern Med. 2024;121:1–3. 10.1016/j.ejim.2023.12.029. 38171934 10.1016/j.ejim.2023.12.029

[11] Koster TM, Wetterslev J, Gluud C, Jakobsen JC, Kaufmann T, Eck RJ, et al. Apparently conclusive meta-analyses on interventions in critical care may be inconclusive-a meta-epidemiological study. J Clin Epidemiol. 2019;114:1–10. 10.1016/j.jclinepi.2019.05.011. 31200004 10.1016/j.jclinepi.2019.05.011

[12] Kang H. Trial sequential analysis: novel approach for meta-analysis. Anesth Pain Med (Seoul). 2021;16(2):138–50. 10.17085/apm.21038. 33940767 10.17085/apm.21038 PMC8107247

[13] Merola R, Iacovazzo C, Troise S, Marra A, Formichella A, Servillo G, et al. Timing of Tracheostomy in ICU Patients: A Systematic Review and Meta-Analysis of Randomized Controlled Trials. Life (Basel). 2024;14(9). 10.3390/life14091165. 10.3390/life14091165 PMC11433256 39337948

[14] Merola R, Vargas M, Marra A, Buonanno P, Coviello A, Servillo G, et al. Videolaryngoscopy versus Fiberoptic Bronchoscopy for Awake Tracheal Intubation: A Systematic Review and Meta-Analysis of Randomized Controlled Trials. J Clin Med. 2024;13(11). 10.3390/jcm13113186. 10.3390/jcm13113186 PMC11173084 38892899

[15] Roshanov PS, Dennis BB, Pasic N, Garg AX, Walsh M. When is a meta-analysis conclusive? A guide to Trial Sequential Analysis with an example of remote ischemic preconditioning for renoprotection in patients undergoing cardiac surgery. Nephrol Dial Transplant. 2017;32(suppl_2):ii23-ii30. 10.1093/ndt/gfw219. 10.1093/ndt/gfw219 28380638

[16] Schünemann HJ, Neumann I, Hultcrantz M, Brignardello-Petersen R, Zeng L, Murad MH, et al. GRADE guidance 35: update on rating imprecision for assessing contextualized certainty of evidence and making decisions. J Clin Epidemiol. 2022;150:225–42. 10.1016/j.jclinepi.2022.07.015. 35934266 10.1016/j.jclinepi.2022.07.015

[17] Borenstein M, Hedges LV, Higgins JP, Rothstein HR. Introduction to meta-analysis. John Wiley & Sons; 2021.

[18] Wetterslev J, Thorlund K, Brok J, Gluud C. Estimating required information size by quantifying diversity in random-effects model meta-analyses. BMC Med Res Methodol. 2009;9:86. 10.1186/1471-2288-9-86. 20042080 10.1186/1471-2288-9-86 PMC2809074

[19] Higgins JP, Whitehead A, Simmonds M. Sequential methods for random-effects meta-analysis. Stat Med. 2011;30(9):903–21. 10.1002/sim.4088. 21472757 10.1002/sim.4088 PMC3107948

[20] Veroniki AA, Jackson D, Viechtbauer W, Bender R, Bowden J, Knapp G, et al. Methods to estimate the between-study variance and its uncertainty in meta-analysis. Res Synth Methods. 2016;7(1):55–79. 10.1002/jrsm.1164. 26332144 10.1002/jrsm.1164 PMC4950030

[21] Langan D, Higgins JP, Simmonds M. An empirical comparison of heterogeneity variance estimators in 12 894 meta-analyses. Res Synth Methods. 2015;6(2):195–205. 10.1002/jrsm.1140. 26053175 10.1002/jrsm.1140

[22] Langan D, Higgins JPT, Simmonds M. Comparative performance of heterogeneity variance estimators in meta-analysis: a review of simulation studies. Res Synth Methods. 2017;8(2):181–98. 10.1002/jrsm.1198. 27060925 10.1002/jrsm.1198

[23] Langan D, Higgins JPT, Jackson D, Bowden J, Veroniki AA, Kontopantelis E, et al. A comparison of heterogeneity variance estimators in simulated random-effects meta-analyses. Res Synth Methods. 2019;10(1):83–98. 10.1002/jrsm.1316. 30067315 10.1002/jrsm.1316

[24] Hansel J, Rogers AM, Lewis SR, Cook TM, Smith AF. Videolaryngoscopy versus direct laryngoscopy for adults undergoing tracheal intubation. Cochrane Database Syst Rev. 2022;4(4):Cd011136. 10.1002/14651858.CD011136.pub3. 10.1002/14651858.CD011136.pub3 PMC8978307 35373840

[25] Deeks JJ, Higgins JP, Altman DG, Group CSM. Analysing data and undertaking meta‐analyses. Cochrane handbook for systematic reviews of interventions. 2019:241–84. 10.1002/9781119536604.ch10.

[26] DerSimonian R, Laird N. Meta-analysis in clinical trials. Control Clin Trials. 1986;7(3):177–88. 3802833 10.1016/0197-2456(86)90046-2

[27] Viechtbauer W. Bias and Efficiency of Meta-Analytic Variance Estimators in the Random-Effects Model. Journal of Educational and Behavioral Statistics. 2005;30(3):261–93. 10.3102/10769986030003261.

[28] Paule RC, Mandel J. Consensus Values and Weighting Factors. J Res Natl Bur Stand. 1982;87(5):377–85. 10.6028/jres.087.022. 10.6028/jres.087.022 PMC6768160 34566088

[29] Sidik K, Jonkman JN. Simple Heterogeneity Variance Estimation for Meta-Analysis. J R Stat Soc: Ser C: Appl Stat. 2005;54(2):367–84. 10.1111/j.1467-9876.2005.00489.x.

[30] Schmidt FL, Hunter JE. Methods of Meta-Analysis: Correcting Error and Bias in Research Findings. Third Edition ed. 55 City Road, London: 2015. 10.4135/9781483398105.

[31] Botta-Dukát Z. Quartile coefficient of variation is more robust than CV for traits calculated as a ratio. Sci Rep. 2023;13(1):4671. 10.1038/s41598-023-31711-8. 36949089 10.1038/s41598-023-31711-8 PMC10033673

[32] Reed GF, Lynn F, Meade BD. Use of coefficient of variation in assessing variability of quantitative assays. Clin Diagn Lab Immunol. 2002;9(6):1235–9. 10.1128/cdli.9.6.1235-1239.2002. 12414755 10.1128/CDLI.9.6.1235-1239.2002 PMC130103

[33] Schwarzer G, Carpenter JR, Rücker G. Meta-analysis with R. Springer; 2015.

[34] Balduzzi S, Rücker G, Schwarzer G. How to perform a meta-analysis with R: a practical tutorial. BMJ Ment Health. 2019;22(4):153–60. 10.1136/ebmental-2019-300117 PMC10231495 31563865

[35] Schwarzer G: meta: General Package for Meta-Analysis. https://CRAN.R-project.org/package=meta (2024). Accessed Jan. 30 2024.

[36] Kang E: smiles: Sequential Method in Leading Evidence Synthesis. https://CRAN.R-project.org/package=smiles (2024). Accessed August 24 2024.

[37] Riberholt CG, Olsen MH, Gluud C. Research Note: Trial sequential analysis in systematic reviews with meta-analysis. J Physiother. 2024;70(3):243–6. 10.1016/j.jphys.2024.05.008. 38908996 10.1016/j.jphys.2024.05.008

[38] Novianti PW, Roes KC, van der Tweel I. Estimation of between-trial variance in sequential meta-analyses: a simulation study. Contemp Clin Trials. 2014;37(1):129–38. 10.1016/j.cct.2013.11.012. 24321246 10.1016/j.cct.2013.11.012

