# Supplementary material for: The conclusiveness of trial sequential analysis varies with estimation of between-study variance: a case study
Source: BMC Med Res Methodol. 2025 Apr 17;25:101. doi: 10.1186/s12874-025-02545-x (PMC12004556; doi:10.1186/s12874-025-02545-x)
Supplement: Supplementary file 1 — Supplementary Material 1. Data source, effect estimates, and assessment of heterogeneity, Equations for between-study variance estimators, Equations for quantities used in trial sequential analysis, TSA software and R package smiles return the same required information size, R code to perform the sequential analysis in this study, Summary of quantities computed in the trial sequential analyses for this study, Flowchart of the steps in using the random-effect model in trial sequential analysis, Scatter plots of the adjustment factor (AF) versus the estimated between-study variance ($\widehat{{\varvec{\tau}}}$τ^2), Descriptive statistics and variability of quantities in the trial sequential analyses. [file 12874_2025_2545_MOESM1_ESM.pdf]

DOI:10.1186/s12874-025-02545-x

SUPPLEMENTARY MATERIAL

The conclusiveness of trial sequential analysis varies with estimation of between-study variance: a case study

Enoch Kang, M.A., consultant[1,2,3]**, James S. Hodges,** Ph.D., Prof.[4,5,6]**,**
Yu-Chieh Chuang, M.D.[7,8]**, Jin-Hua Chen,** Ph.D., Prof.[1,9,10,11]**,**
Chiehfeng Chen, M.D., Ph.D., Prof.[1,2,12,13,*]**, Cochrane Taiwan**[#]

1. Cochrane Taiwan, Taipei Medical University, Taipei, Taiwan
2. Evidence-Based Medicine Center, Wan Fang Hospital, Taipei Medical University, Taipei, Taiwan
3. Institute of Health Policy & Management, College of Public Health, National Taiwan University, Taipei, Taiwan
4. Division of Biostatistics and Health Data Sciences, School of Public Health, University of Minnesota, Minneapolis, Minnesota, USA
5. Graduate Institute of Data Science, College of Management, Taipei Medical University, Taipei, Taiwan
6. College of Public Health, National Taiwan University, Taipei, Taiwan.
7. Department of Psychiatry, Taipei City Psychiatric Center, Taipei City Hospital, Songde branch, Taipei, Taiwan
8. School of Medicine, College of Medicine, Taipei Medical University, Taipei, Taiwan
9. Graduate Institute of Data Science, College of Management, Taipei Medical University, Taipei 110, Taiwan.
10. Research Center of Biostatistics Center, College of Management, Taipei Medical University, Taipei 110, Taiwan.
11. Biostatistics Center, Wan Fang Hospital, Taipei Medical University, Taipei 116, Taiwan.
12. Department of Public Health, School of Medicine, College of Medicine, Taipei Medical University, Taipei, Taiwan
13. Division of Plastic Surgery, Department of Surgery, Wan Fang Hospital, Taipei Medical University, Taipei, Taiwan

[#] Group author:

Professor, **Ka-Wai Tam**, M.D., Ph.D., Professor, **Kee-Hsin Chen**, Ph.D., **Wen Hsuan Hou**, M.D., Ph.D., Professor, **Tsai-Wei Huang**, Ph.D., Professor, **El-Wui Loh**, Ph.D.

* Correspondence:
Chiehfeng Chen, M.D., Ph.D., Prof.
Evidence-based Medicine Center, Wan Fang Hospital, Taipei Medical University
No. 111, Section 3, Xinglong Road, Taipei 116, Taiwan
E-mail: clifchen@tmu.edu.tw
Telephone number: +886-2-29307930 ext. 7220

Content

Supplementary file 1. Data source, effect estimates, and assessment of heterogeneity

Supplementary file 2. Equations for between-study variance estimators

Supplementary file 3. Equations for quantities used in trial sequential analysis

Supplementary file 4. TSA software and R package *smiles* return the same required information size

Supplementary file 5. R code to perform the sequential analysis in this study

Supplementary file 6. Summary of quantities computed in the trial sequential analyses for this study

Supplementary file 7. Flowchart of the steps in using the random-effect model in trial sequential analysis.

Supplementary file 8. Scatter plots of the adjustment factor (AF) versus the estimated between-study variance ($\hat{\tau}^2$)

Supplementary file 9. Descriptive statistics and variability of quantities in the trial sequential analyses

Supplementary file 1. Data source, effect estimates, and assessment of heterogeneity

**Information**	**Hypoxaemia**	**Failure (difficulty)**	**Failure (expert)**	**Failure (non-obese)**
Data source [a]	Analysis 3.2	Analysis 1.13.1	Analysis 4.8.1	Analysis 4.6.2
Risk ratio	0.25	0.37	0.41	0.47
95% CI	0.12 to 0.50	0.19 to 0.74	0.33 to 0.50	0.32 to 0.56
τ^2 based on DL	0.00	0.35	0	0.33
χ^2 for the heterogeneity test	4.01	13.39	42.74	105.68
df for the heterogeneity test	7	8	47	61
P of the heterogeneity test	0.68	0.0993	0.65	0.0004
I^2	0%	40%	0%	39%

[a] Data from this Cochrane review: Hansel J, Rogers AM, Lewis SR, Cook TM, Smith AF. Videolaryngoscopy versus direct laryngoscopy for adults undergoing tracheal intubation. Cochrane Database Syst Rev. 2022;4(4):Cd011136. doi: 10.1002/14651858.CD011136.pub3.

Supplementary file 2. Equations for between-study variance estimators

Basic 1. The variance of the combined effect in the fixed-effect model is the reciprocal of the sum of the weights:

$$v_{fixed} = \frac{1}{\sum_{i=1}^{j} W_i} \quad \ldots(1)$$

where
i indexes individual studies.
j is the total number of studies.
W_i is the weight for study i.

Basic 2. The simple weight for each study is the reciprocal of the within-study variance:

$$W_i = \frac{1}{v_i} \quad \ldots(2)$$

where
v_i is the within-study variance for study i.

Basic 3. The weight for each study in the random-effects model is the reciprocal of the sum of within-study variance and between-study variance:

$$W_{random.i} = \frac{1}{v_i + \hat{\tau}^2} \quad \ldots(3)$$

where
v_i is the within-study variance for study i.
$\hat{\tau}^2$ is the between-study variance in random-effects model.

Basic 4. The generalized Q-statistic is calculated for the total variance:

$$Q = \sum_{i=1}^{j} w_i \left(\theta_i - \hat{\theta}\right)^2 \quad \ldots(4)$$

where
i indexes individual studies.
j is the total number of studies.
W_i is the weight for study i.
θ_i is the effect of study i.
$\hat{\theta}$ is the weighted effect.

Basic 5. A scaling factor, C, is required because Q is a weighted sum of squares.

$$C = \sum_{i=1}^{j} W_i - \frac{\sum_{i=1}^{j} {w_i}^2}{\sum_{i=1}^{j} w_i} \quad \ldots(5)$$

where
i indexes individual studies.
j is the total number of studies.
W_i is the weight for study i.

Main 1. Between-study variance by DerSimonian-Laird's method ($\hat{\tau}^2_{DL}$) is:

$$\hat{\tau}^2_{DL} = \begin{cases} \frac{Q - df}{C}, & if\ Q > df \\ 0, & if\ Q \le df \end{cases} \quad \ldots(6)$$

where
C is the scaling factor.
df degree of freedom for the pooled analysis.
Q is the total number of studies.

An equivalent formula for computing is:

$$\hat{\tau}^2_{DL} = \frac{\sum_{i=1}^{j} w_i \left(\theta_i - \hat{\theta}\right)^2 - (j-1)}{\sum_{i=1}^{j} w_i - \frac{\sum_{i=1}^{j} w_i^2}{\sum_{i=1}^{j} w_i}} \quad \ldots(7)$$

where
i indexes individual studies.
j is the total number of studies.
w_i is the weight for study i.
θ_i is the effect of study i.
$\hat{\theta}$ is the weighted effect.

Main 2. Between-study variance by restricted maximum-likelihood ($\hat{\tau}^2_{REML}$) is:

$$\hat{\tau}^2_{REML} = \frac{\sum_{i=1}^{j} w_{REML.i}^2 \left(\left(\theta_i - \hat{\theta}_{ML}\right)^2 - v_i\right)}{\sum_{i=1}^{j} w_{REML.i}^2} + \frac{1}{\sum_{i=1}^{j} w_{REML.i}} \quad \ldots(8)$$

where
i indexes individual studies.
j is the total number of studies.
v_i is the within-study variance for study i.
$w_{REML.i}$ is the weight for study i in the random-effects based on the reciprocal of the sum of within-study variance (v_i) and estimated between-study variance ($\hat{\tau}^2_{REML}$).
θ_i is the effect of study i.
$\hat{\theta}_{ML}$ is the weighted effect using maximum likelihood approach.

Main 3. Between-study variance by Paule-Mandel's method ($\hat{\tau}^2_{PM}$) is:

$$\hat{\tau}^2_{PM} = \frac{Q - \sum_{i=1}^{j} w_{PM.i} \times v_i + \frac{\sum_{i=1}^{j} w_{PM.i} \times v_i}{\sum_{i=1}^{j} w_{PM.i}}}{\sum_{i=1}^{j} w_{PM.i} - \frac{\sum_{i=1}^{j} w_{PM.i}^2}{\sum_{i=1}^{j} w_{PM.i}}} \quad \ldots(9)$$

where
i indexes individual studies.
j is the total number of studies.
v_i is the within-study variance for study i.
Q is the generalized Q-statistic.
$w_{PM.i}$ is the weight for study i in the random-effects based on the reciprocal of the sum of within-study variance (v_i) and estimated between-study variance ($\hat{\tau}^2_{PM}$).

Main 4. Between-study variance by maximum-likelihood ($\hat{\tau}^2_{ML}$) is:

$$\hat{\tau}^2_{ML} = \frac{\sum_{i=1}^{j} w_{ML.i}{}^2 \left(\left(\theta_i - \hat{\theta}_{ML} \right)^2 - v_i \right)}{\sum_{i=1}^{j} w_{ML.i}{}^2} \quad \ldots(10)$$

where

i indexes individual studies.

j is the total number of studies.

v_i is the within-study variance for study *i*.

$w_{ML.i}$ is the weight for study i in the random-effects model based on the reciprocal of the sum of within-study variance (v_i) and estimated between-study variance ($\hat{\tau}^2_{ML}$).

θ_i is the effect of study *i*.

$\hat{\theta}_{ML}$ is the weighted effect using maximum likelihood approach.

Main 5. Between-study variance by Sidik-Jonkman's method ($\hat{\tau}^2_{SJ}$) is:

$$\hat{\tau}^2_{SJ} = \frac{1}{J-1} \sum_{i=1}^{j} w_{SJ.0.i} \left(\theta_i - \hat{\theta}_{SJ.0} \right)^2 \quad \ldots(11)$$

where

i indexes individual studies.

j is the total number of studies.

$w_{SJ.0.i}$ is the weight for study i in the random-effects model based on the method proposed by Sidik and Jonkman (((v_i / $\hat{\tau}^2_0$) – 1)$^{-1}$).

$\hat{\tau}^2_0$ is the initial between-study variance using the method proposed by Sidik and Jonkman ($\sum_{i=1}^{j} \left(\theta_i - \sum_{i=1}^{j} \frac{\theta_i}{j} \right)^2$)

θ_i is the effect of study *i*.

$\hat{\theta}_{SJ.0}$ is the initial weighted effect using the method proposed by Sidik and Jonkman ($\sum_{i=1}^{j} W_{SJ.0.i} \times \frac{\theta_i}{\sum_{i=1}^{j} w_{SJ.0.i}}$).

Main 6. Between-study variance by Hunter-Schmidt's method ($\hat{\tau}^2_{HS}$) is defined as follow:

$$\hat{\tau}^2_{HS} = \frac{Q - j}{\sum_{i=1}^{j} w_{HS.i}} \quad \ldots(12)$$

where

i indexes individual studies.

j is the total number of studies.

$W_{HS.i}$ is the weight for study *i* based on the reciprocal of total sample size in the study *i*.

Supplementary file 3. Equations for quantities used in trial sequential analysis

1. The estimated required information size ($\widehat{RIS}$) of a meta-analysis using random-effects model can be expressed as follows:

$$\widehat{RIS} = 4 * \frac{(Z_{\alpha/2}+Z_{\beta})^2 * v_{random}}{\mu^2} \quad \ldots(13)$$

where
α is the predefined overall probability of a false positive.
β is the predefined overall probability of a false negative.
μ^2 is the expected effect.
v_{random} is the variance of pooled effects of the meta-analysis in random-effects model.
Note:
1. v_{random} can be obtained from $\frac{1}{\sum_{i=1}^{j} W_{random.i}}$, and the $W_{random.i}$ is presented in **Equation 3**.
2. $\widehat{RIS}$ here is also unadjusted required information size ($\widehat{RIS}_{unadjusted}$)

2. The estimated diversity ($\widehat{D}^2$) of a meta-analysis using random-effects model can be expressed as follows:

$$\widehat{D}^2 = \frac{v_{random} - v_{fixed}}{v_{random}} \quad \ldots(14)$$

where
v_{fixed} is the variance of pooled effects of the meta-analysis in fixed-effect model.
v_{random} is the variance of pooled effects of the meta-analysis in random-effects model.

An alternative expression of $\widehat{D}^2$ can highlight the role of the estimated between-study variance in the calculation, as follows:

$$\widehat{D}^2 = \frac{1}{\hat{\tau}^2} * (\hat{\tau}^2 + \frac{\hat{\tau}^2 * v_{fixed}}{v_{random} - v_{fixed}}) \quad \ldots(15)$$

where
v_{fixed} is the variance of pooled effects of the meta-analysis in fixed-effect model.
v_{random} is the variance of pooled effects of the meta-analysis in random-effects model.
$\hat{\tau}^2$ is estimated between-study variance of the meta-analysis in random-effects model.

3. Diversity-based adjustment factor ($\widehat{AF}$) for estimating required information size of a meta-analysis using random-effects model can be expressed as follows:

$$\widehat{AF} = \frac{1}{(1-\widehat{D}^2)} \quad \ldots(16)$$

where
$\widehat{D}^2$ is the estimated diversity in the random-effects meta-analysis, as given in **Equation 15**.

An alternative expression of $\widehat{AF}$ can highlight the role of the estimated between-study variance in the calculation, as follows:

$$\widehat{AF} = [1 - \frac{1}{\hat{\tau}^2} * (\hat{\tau}^2 + \frac{\hat{\tau}^2 * v_{fixed}}{v_{random} - v_{fixed}})]^{-1} \quad \ldots(17)$$

where
v_{fixed} is the variance of pooled effects of the meta-analysis in fixed-effect model.
v_{random} is the variance of pooled effects of the meta-analysis in random-effects model.
$\hat{\tau}^2$ is estimated between-study variance of the meta-analysis in random-effects model.

4. The adjusted required information size ($\widehat{RIS}_D{}^2$) of a meta-analysis using random-effects model can be expressed as follows:

$$\widehat{RIS}_D{}^2 = \widehat{RIS}_{unadjusted} \times \widehat{AF} \qquad \ldots(18)$$

where

$\widehat{AF}$ is the diversity-based adjustment factor, as given in **Equation 16 and 17**.

$\widehat{RIS}_{unadjusted}$ is the unadjusted required information size, as given in **Equation 13**.

An alternative expression of $\widehat{RIS}_D{}^2$ can highlight the role of the estimated between-study variance in the calculation, as follows:

$$\widehat{RIS}_D{}^2 = 4 * \frac{(Z_{\alpha/2}+Z_{\beta})^2 * [\sum_{i=1}^{j}(v_i+\hat{\tau}^2)]^{-1}}{\mu^2} * [1 - \frac{1}{\hat{\tau}^2} * (\hat{\tau}^2 + \frac{\hat{\tau}^2 * v_{fixed}}{v_{random}-v_{fixed}})]^{-1} \qquad \ldots(19)$$

where

α is the predefined overall probability of a false positive.

β is the predefined overall probability of a false negative.

μ^2 is the expected effect.

v_i is the within-study variance for study i.

v_{fixed} is the variance of pooled effects of the meta-analysis in fixed-effect model.

v_{random} is the variance of pooled effects of the meta-analysis in random-effects model.

$\hat{\tau}^2$ is estimated between-study variance of the meta-analysis in random-effects model.

5. According to O'Brien and Fleming's method, α-spending monitoring boundaries in trial sequential analysis can be derived as follows:

$$\alpha_{\widehat{FII}i} = \begin{cases} 0, & if\ \widehat{FII}_i = 0 \\ 2 - 2\Phi\left(\frac{Z_{\frac{1}{2}\alpha}}{\sqrt{\widehat{FII}i}}\right), & if\ 0 < \widehat{FII}_i \le 1 \end{cases} \qquad \ldots(20)$$

where

α is the predefined overall probability of a false positive.

$\widehat{FII}$ is fraction of information index for determining the α-spending monitoring boundaries.

$\widehat{FII}_i$ represents check points of the sequential analysis.

$\alpha_{\widehat{FII}i}$ is the cumulative probability of a false positive on each check point.

Φ is the standard normal distribution function.

When $\widehat{FII}_i$ reaches 1, α is the predetermined Type I error.

Supplementary file 4. TSA software and R package *smiles* return the same required information size

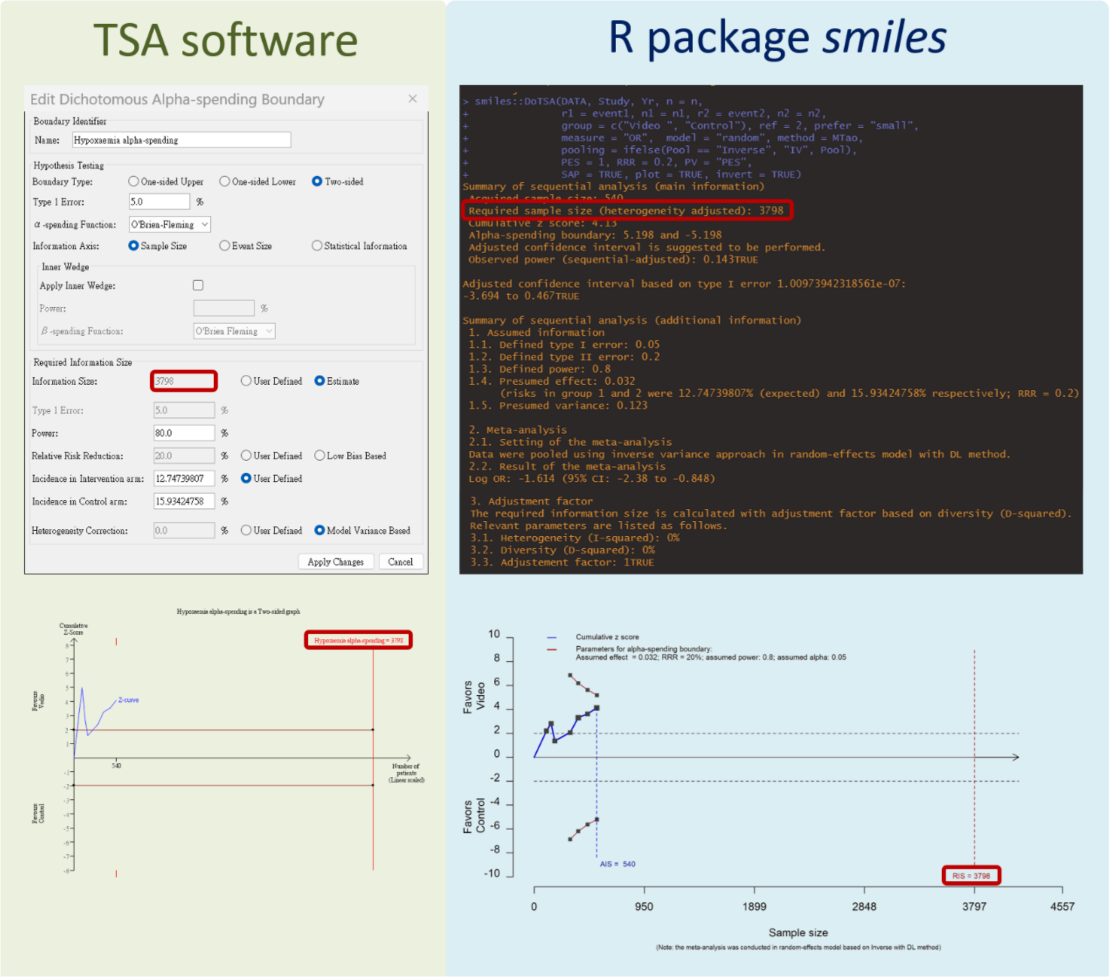

Supplementary file 5. R code to perform the sequential analysis in this study

```
# DATA.Org is a long format data set with seven columns, including
   ## Study
   ## id
   ## Yr
   ## event1
   ## n1
   ## event2
   ## n2

# Definition of columns
   ## column 'Study' is the surname of the first author
   ## column 'id' is the sequence number of the study
   ## column 'Yr' is publication year
   ## column 'event1' is event in video group
   ## column 'n1' is sample size in video group
   ## column 'event2' is event in direct group
   ## column 'n2' is sample size in direct group

# Load library
library("meta")
library("smiles")

# Calculate relevant quantities
   ## Step 1: Data preparation
      ### Step 1.1. Load data of each outcome
      ### Step 1.2. Name the outcome
outcomeName <- "1. Hypoxaemia"
                    # "1. Hypoxaemia" | "2. Failure (difficult)"
                    # "3. Failure (expert)" | "4. Failure (non-obese)"
      ### Step 1.3. Extract data for analysis (removing studies with zero cells in both groups)
DATA.Org $cln <- ifelse(DATA.Org$event1 + DATA.Org$event2 == 0, "remove", "keep")
DATA <- DATA.Org [which(DATA.Org [, "cln"] == "keep"), ]

   ## Step 2: Conduct meta-analysis and sequential analysis with a specific between-study variance
estimators and pooling method
for (MTao in c("DL", "REML", "PM", "SJ", "HS", "ML")) {
      MA.RE <- meta::metabin(data = DATA,
                              studlab = Study,
                              event.e = event1,
                              n.e = n1,
                              event.c = event2,
                              n.c = n2,
                              comb.random = TRUE,
                              comb.fixed = FALSE,
                              method.tau = MTao,
                              method = "Inverse",
                              sm = "OR")

      rsltTSA <- smiles::DoTSA(DATA,
                               Study,
                               Yr,
                               n = n,
                               r1 = event1,
                               n1 = n1,
```

```
                                    r2 = event2,
                                    n2 = n2,
                                    group = c("Video ", "Control"),
                                    ref = 2,
                                    prefer = "small",
                                    measure = "OR",
                                    model = "random",
                                    method = MTao,
                                    pooling = "IV",
                                    PES = 1,
                                    RRR = 0.2,
                                    PV = "PES",
                                    SAP = TRUE)

  ## Step 3: Tabulate relevant information and quantities
    if (MTao == "DL") {
      tblTSA <- data.frame(outcome = outcomeName,
                                  model = MTao,
                                  pool = "IV",
                                  AIS = rsltTSA$AIS,
                                  Tau2 = MA.RE$tau2,
                                  D2 = rsltTSA$diversity,
                                  AF = rsltTSA$AF,
                                  RIS = rsltTSA$RIS.adj,
                                  zReq = rsltTSA$data[nrow(rsltTSA$data) -
sum(is.na(rsltTSA$data$source)), "asub"],
                                  zCum = rsltTSA$data[nrow(rsltTSA$data) -
sum(is.na(rsltTSA$data$source)), "zCum"])
    } else {
      tblTSATemp <- data.frame(outcome = outcomeName,
                                      model = MTao,
                                      pool = "IV",
                                      AIS = rsltTSA$AIS,
                                      Tau2 = MA.RE$tau2,
                                      D2 = rsltTSA$diversity,
                                      AF = rsltTSA$AF,
                                      RIS = rsltTSA$RIS.adj,
                                      zReq = rsltTSA$data[nrow(rsltTSA$data) -
sum(is.na(rsltTSA$data$source)), "asub"],
                                      zCum = rsltTSA$data[nrow(rsltTSA$data) -
sum(is.na(rsltTSA$data$source)), "zCum"])
      tblTSA <- rbind(tblTSA, tblTSATemp)
  }
}
```

Supplementary file 6. Summary of quantities computed in the trial sequential analyses for this study

outcome	model	AIS	Tau2	D2	AF	RIS	zReq	zCum
1. Hypoxaemia	DL	540	0.00	0.00	1.00	3798	-5.20	-4.13
1. Hypoxaemia	REML	540	0.00	0.00	1.00	3798	-5.20	-4.13
1. Hypoxaemia	PM	540	0.00	0.00	1.00	3798	-5.20	-4.13
1. Hypoxaemia	SJ	540	0.59	0.43	1.75	6655	-6.88	-3.14
1. Hypoxaemia	HS	540	0.00	0.00	1.00	3798	-5.20	-4.13
1. Hypoxaemia	ML	540	0.00	0.00	1.00	3798	-5.20	-4.13
2. Failure (difficult)[a]	DL	1113	0.15	0.51	2.05	9088	-5.60	-3.21
2. Failure (difficult)[a]	REML	1113	0.27	0.62	2.64	11719	-6.36	-3.13
2. Failure (difficult)[a]	PM	1113	0.10	0.43	1.76	7805	-5.19	-3.25
2. Failure (difficult)[a]	SJ	1113	0.49	0.71	3.48	15477	-7.31	-2.99
2. Failure (difficult)[a]	HS	1113	0.04	0.25	1.34	5940	-4.53	-3.32
2. Failure (difficult)[a]	ML	1113	0.13	0.48	1.91	8495	-5.41	-3.23
3. Failure (expert)	DL	6148	0.00	0.00	1.00	8254	-2.27	-8.23
3. Failure (expert)	REML	6148	0.03	0.14	1.16	9613	-2.45	-7.53
3. Failure (expert)	PM	6148	0.00	0.00	1.00	8254	-2.27	-8.23
3. Failure (expert)	SJ	6148	0.75	0.64	2.81	23190	-3.81	-4.90
3. Failure (expert)	HS	6148	0.00	0.00	1.00	8254	-2.27	-8.23
3. Failure (expert)	ML	6148	0.01	0.04	1.04	8611	-2.32	-8.03
4. Failure (non-obese)	DL	8271	0.37	0.49	1.96	17017	-2.81	-5.56
4. Failure (non-obese)	REML	8271	0.27	0.44	1.77	15399	-2.67	-5.86
4. Failure (non-obese)	PM	8271	0.56	0.56	2.28	19791	-3.03	-5.16
4. Failure (non-obese)	SJ	8271	1.33	0.71	3.41	29629	-3.71	-4.29
4. Failure (non-obese)	HS	8271	0.33	0.47	1.88	16326	-2.75	-5.68
4. Failure (non-obese)	ML	8271	0.22	0.40	1.68	14588	-2.60	-6.03
5. Failure (difficult)[b]	DL	1144	0.40	0.62	2.64	7492	-5.02	-4.39
5. Failure (difficult)[b]	REML	1144	0.14	0.42	1.74	4924	-4.07	-5.60
5. Failure (difficult)[b]	PM	1144	0.81	0.73	3.75	10637	-5.98	-3.64
5. Failure (difficult)[b]	SJ	1144	1.58	0.82	5.56	15750	-7.27	-2.99
5. Failure (difficult)[b]	HS	1144	0.22	0.52	2.07	5859	-4.44	-5.05
5. Failure (difficult)[b]	ML	1144	0.02	0.14	1.16	3287	-3.32	-7.22

[a.] data based on the trials comparing Macintosh-style video laryngoscopy to direct laryngoscopy;

[b.] data based on the trials comparing hyper-angulated video laryngoscopy to direct laryngoscopy.

AF, adjustment factor; AIS, acquired information size; D2, D^2 is diversity; DL, DerSimonian-Laird estimator; HS, Hunter-Schmidt estimator; MH, Mantel-Haenszel; ML, Maximum-likelihood estimator; PM, Paule-Mandel estimator; REML, Restricted maximum-likelihood estimator; RIS, required information size; SJ, Sidik-Jonkman estimator; Tau2, τ^2 is between-study variance; zCum, observed cumulative z-score; zReq, z-score of α-spending monitoring boundary.

DOI:10.1186/s12874-025-02545-x

Supplementary file 7. Flowchart of the steps in using the random-effect model in trial sequential analysis.

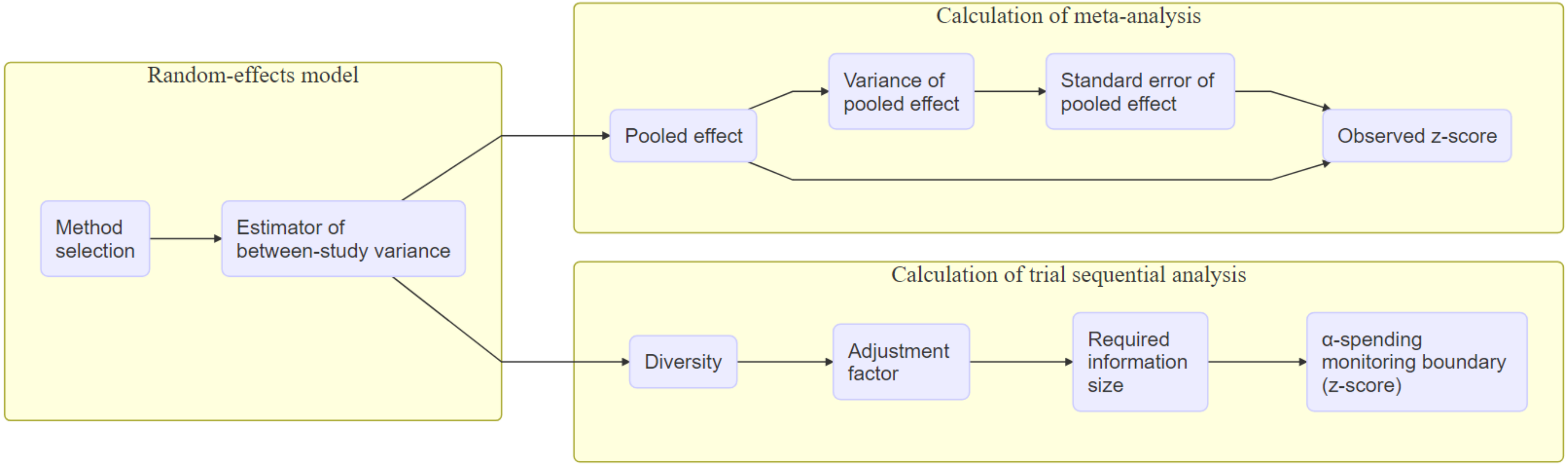

Supplementary file 8. Scatter plots of the adjustment factor (AF) versus the estimated between-study variance ($\hat{\tau}^2$)

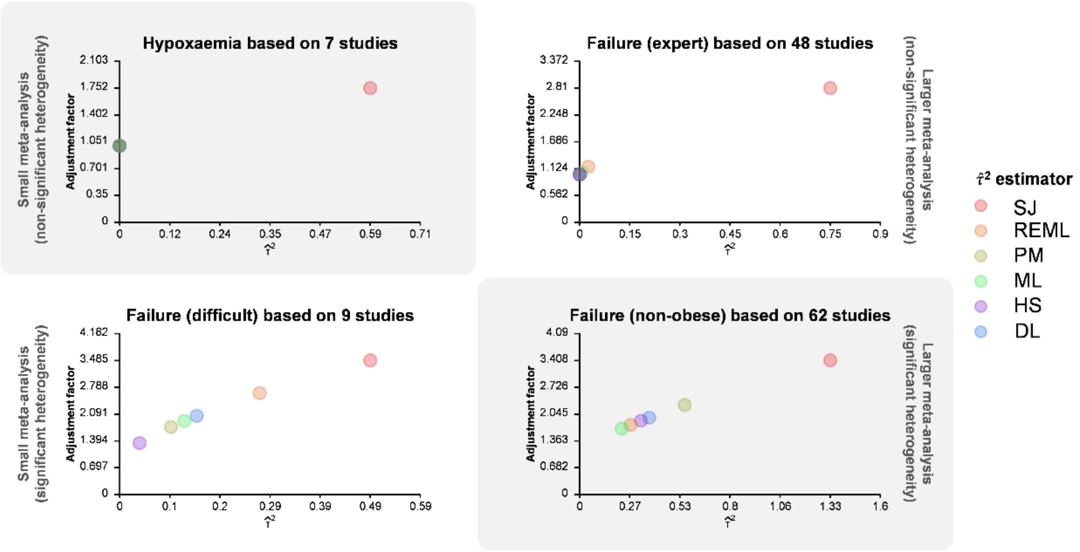

DL, DerSimonian-Laird estimator; HS, Hunter-Schmidt estimator; ML, Maximum-likelihood estimator; PM, Paule-Mandel estimator; REML, Restricted maximum-likelihood estimator; SJ, Sidik-Jonkman estimator.

Supplementary file 9. Descriptive statistics and variability of quantities in the trial sequential analyses

Basic information and further metrics	Hypoxaemia	Failure (difficulty)[a]	Failure (expert)	Failure (non-obese)
**Diversity**				
Minimum	0.00	0.25	0.00	0.40
The first quartile	0.00	0.44	0.00	0.44
Median	0.00	0.49	0.02	0.48
The third quartile	0.00	0.59	0.12	0.54
Maximum	0.43	0.71	0.64	0.71
QCV	N/A[b]	15%	100%	10%
**Adjustment factor**				
Minimum	1.00	1.34	1.00	1.68
The first quartile	1.00	1.80	1.00	1.80
Median	1.00	1.98	1.02	1.92
The third quartile	1.00	2.49	1.13	2.20
Maximum	1.75	3.48	2.81	3.41
QCV	0%	16%	6%	10%
**Required information size**				
Minimum	3798	5940	8254	14588
The first quartile	3798	7978	8254	15631
Median	3798	8792	8433	16672
The third quartile	3798	11061	9363	19098
Maximum	6655	15477	23190	29629
QCV	0%	16%	6%	10%
**α-spending monitoring boundaries**				
Minimum	-6.88	-7.31	-3.81	-3.71
The first quartile	-5.20	-6.17	-2.42	-2.98
Median	-5.20	-5.51	-2.30	-2.78
The third quartile	-5.20	-5.25	-2.27	-2.69
Maximum	-5.20	-4.53	-2.27	-2.60
QCV	0%	8%	3%	5%
**Observed cumulative z-score**				
Minimum	-4.13	-3.32	-8.23	-6.03
The first quartile	-4.13	-3.25	-8.23	-5.81
Median	-4.13	-3.22	-8.13	-5.62
The third quartile	-4.13	-3.15	-7.65	-5.26
Maximum	-3.14	-2.99	-4.90	-4.29
QCV	0%	2%	4%	5%

[a]. data based on the trials comparing Macintosh-style video laryngoscopy to direct laryngoscopy.
[b]. Diversity is 0.
QCV, Quartile coefficient of variation
